# What Value Has the Microscope in Examinations for Gonorrhea?1Read before the Journal Club, January 26, 1894.

**Published:** 1894-03

**Authors:** A. Neisser, G. A. Himmelsbach

**Affiliations:** 137 West Tupper Street; Breslau; Clinical Instructor in Medicine, University Dispensary, Buffalo, N. Y.


### WHAT VALUE HAS THE MICROSCOPE IN EXAMINATIONS FOR GONORRHEA?[1]

By PROF. A. NEISSER, Breslau.

(Translated from the *Deutsche Medicinische Wochenschrift*, Nos. 29–30, 1893.)

By G. A. HIMMELSBACH, M. D., Buffalo, N. Y.,
Clinical Instructor in Medicine, University Dispensary, Buffalo, N. Y.

Dr. Broese (Berlin), in his lecture before the Society of Obstetrics and Gynecology, at Berlin, upon The Etiology, Diagnosis and Therapy of Gonorrhea in Women, made certain statements which I consider should not be allowed to pass unanswered, particularly so because none of those who took part in the discussion opposed him. Such views, even though supported by other eminent men—for instance, B. Sänger—are entirely contrary to those entertained by me and my friends for several years past. Their views appear to me, in every respect, to be so very poorly grounded and proven, that their general acceptance hereafter will prove dangerous to all questions concerning gonorrhea, especially as regards its prophylaxis and therapeutics.

I, therefore, feel myself called upon to illustrate once more our principles of treating gonorrhea, in opposition to those advocated by Dr. Broese. The differences are briefly stated as follows: A general use of the microscope, to prove the presence or absence of the gonococcus, is unquestionably the best and, undoubtedly, the most efficient means at our command. He who does not use this method is not only making a great mistake, but also deprives himself of important advantages. In view of the fact that this

1. Read before the Journal Club, January 26, 1894.

method is often both difficult and imperfect, it requires repeated examinations, and those who make use of this means require considerable experience.

What do our opponents say ?

This method of the use of the microscope to detect the gonococcus is of positive value *only* in case of doubt (or error), besides we have other methods in abundance ; then, too, it is so little to be depended upon that we prefer to do away with it entirely.

Such a standpoint as this ought to be fought against. It is most astonishing to hear Dr. Broese clearly express his conviction that the gonococcus is the cause of all gonorrheal processes.

If that is the case, then we must admit the practical value of the microscope in order to study the many characteristics and properties of the bacteria, and also its practical use in the diagnosis and treatment, besides the satisfaction of a surer therapeutic success in all the stages of treatment. If there exists any difference of opinion as regards its usefulness, it is mainly in the methods of examination.

Now, then, wherein does my acknowledgment of the usefulness and the necessity of examining for the gonococcus consist ?

In innumerable instances, in which we could neither by macroscopic inspection nor a most careful clinical investigation state with absolute certainty whether the discharge from the mucous membrane is a specific gonorrhea, due to the presence of the gonococcus, or whether it is nothing but the result of an inflammation, due to other causes, the microscope will detect the presence of the gonococcus, and then a positive diagnosis is established. With a positive diagnosis, we are enabled to undertake the proper treatment of our patients, with due regard as to etiology. Instances without number in which men, who are about to enter matrimony, present themselves at my office, brought there by a delicate feeling of foresight. A thorough, day after day, microscopic examination of the urethral mucus, the urinary shreds, and the prostatic secretions, etc., generally results in the proof of the existence of the gonococcus.

The diagnosis in women is, undoubtedly, much more difficult ; nevertheless, a great many cases have been successfully examined and a positive diagnosis arrived at. Hundreds of prostitutes are annually snatched from their disorderly, vagabond life, and placed in a hospital for the care of such cases, although regularly examined by one of the district physicians and pronounced sound in body. These very women would have diseased all those who had intercourse with them. Furthermore, how often do we discover, by means of the microscope, even in married women, the undoubted existence of gonorrhea, and thereby establish a foundation for further therapy?

We should always be loth to set aside a microscopic investigation for the gonococcus, which is conclusive, even though the discharge is so diminutive that it is hardly noticed, or even if the objective symptoms of the disease are wanting; or, again, even in quite simple cases, where the urethral discharge is profuse.

In the first place, there is the fact (all mechanical causes excluded) that there are cases of urethritis procreated by other bacteria. It is true that such cases are rare, but, nevertheless, they are sometimes met with. The positive diagnosis, in such cases, will materially influence the prognosis and therapeutics. I am treating, at the present time, a gentleman (county judge), married for the past nine years, who under oath declares that during this time he has not had coitus with any woman except his wife. He first consulted me in February of this year, and, upon inspection, discovered what I took to be simple gonorrhea; but, upon repeated inspection and examination, I was unable to find the gonococcus, yet found the discharge mingled with immense quantities of diplococci, presenting themselves intra- and extracellular, apparently arranged in one long string. Under treatment this condition disappeared quite rapidly. Since then my patient has had a recurrence of the same soon after intercourse with his wife. I regret to say that I have not yet had an opportunity to examine his wife, to prove whether every new infection comes through her.

A second but more common appearance of gonorrhea, in men and women, is where they have previously had an undisputed attack of the disease and had apparently been cured; then, subsequently, through cohabitation, contracted the same disease, or, at least, set up an acute purulent discharge. In all such cases, microscopic examinations will result in the following conclusions: (1) Has there entered a fresh (new) infection? Or (2) is it a recrudescence of an old, latent, inflammatory process of a gonorrheal nature, even though all traces of gonorrhea had long since been removed?

Now, then, the question is: Is there a fresh infection or a revival of the old gonorrheal processes? This cannot be decided with certainty, because the difference in the number of gonococci in both cases is not so strikingly great that a safe decision is warranted. Very often, however, we have to do with cases where there is considerable pus without the presence of the gonococcus. This condition usually subsides in a few days by a simple, mild, nonspecific, that is to say, non-parasitic treatment, while newly contracted infections will take on a chronic course. Again, there are cases of exacerbations of gonorrhea (gonococcus) in which the clinical phenomena disappear very early. In these instances, a differential diagnosis cannot be made with surety, from the progress of the disease alone, without an examination for the gonococcus.

Further explanation is unnecessary as to the importance of fixing this differential diagnosis at once, both as regards its relation to prognosis and therapeutics, besides, also, from a humanitarian standpoint. I must not omit to report the following case which I had an opportunity of seeing a year ago:

The patient, an officer, consulted me first in August, 1888; eleven months prior to that time he suffered from acute gonorrhea, accompanied by left epididymitis. At a more previous date than that he is said to have suffered pain in his right epididymis. Still, at the time of his consultation, there were no manifest changes present.

Often repeated examinations did not reveal any sign of secretion or any flakes; all subjective symptoms were wanting. In February, 1890, this patient, who had then been married eighteen months, returned to me. Both he and his wife felt perfectly well. His wife was examined gynecologically and pronounced healthy; still he wished to be informed whether his former troubles accounted for the barrenness.

Closer examinations showed a well-preserved potentia cöeundi. He said his wife was very easily excited and very often wanted coitus, which he promptly satisfied. The examinations brought to light the following facts: Besides a small varicocele, which was discovered very early, were found nodules attached to a thickened epididymis. Examination of the spermatic fluid showed the spermatozoa completely wanting. I saw the patient quite often after that. I made some effort to remove these nodules and thickening, but without any beneficial result. In June, 1891, my patient came to me in despair, stating that his wife had been off on a visit for two weeks, and when she returned he found himself sick again with gonorrhea, and only after several copulations with her. He said his wife could hardly go a day without several copulations and that she must have become diseased during her absence. He was convinced that divorce proceedings had to follow. The examination disclosed a profuse purulent discharge, which appeared two days after copulation, and he firmly asserted that he had not had intercourse with another woman. I was persuaded that his present attack must have been contracted from his wife. An acquaintance with the case for three years, strengthened by repeated examinations, led me to exclude urethritis.

For the sake of protection and as an expert to have the necessary proofs at hand in view of the divorce proceedings about to be instituted, I made microscopic examinations and preparations. The examinations, however, disclosed great quantities of pus corpuscles, together with a few epithelial cells, but absolutely no gonococci, and, indeed, there was an absence of any bacteria. This investigation was continued daily for two months ; the discharge gradually grew less, though again and again it was examined, but always with the same result, until at last all investigation was stopped by the absence of any flakes. One of my friends and colleagues who generally treated the patient, also saw the discharge and he was just as firmly convinced as myself that it was a typical case of gonorrhea. Our diagnosis, however, was at once rectified upon a microscopic examination. This procedure was also necessary on account of the psychical condition of the patient. It can be safely said that without the use of the microscope the diagnosis of gonorrhea would have been made. The progress of the case was somewhat tedious and accompanied by a mild cystitis ; the latter symptom would also tend to strengthen the diagnosis of gonorrhea. The causes of this acute suppuration I am at a loss to account for, because I could find no other bacteria ; but gonorrhea or any other infection of that man by his wife was all out of the question.

To be sure, not every case we meet presents such dramatic results as the one before us ; but he who meets with many cases of gonorrhea, accompanied by other complications, particularly so when from the better class of society suffering from chronic gonorrhea, must confess how complicated these important and responsible steps become. We must also admit the inestimable value to the physician when he can say that the microscope has shown him the presence of the gonococcus when none were expected; then, again, when an apparently serious discharge has been found to be a harmless non-infectious material.

With women, the diagnosis of all these conditions is much more difficult than with men, and false conclusions with the microscope will happen more often; nevertheless a goodly proportion of cases will present negative symptoms of the disease sufficient to guide him in a correct prognosis and therapy. But gynecologists (E. Fränkel and Sänger), two of my respected friends, oppose me in the following words: "How seldom you andrologists are able to find any gonococci in the husband who contracts the disease from his wife, who suffers from gonorrhea." Well, I concede that not in all cases where there exists these conditions between man and wife do we find a satisfactory explanation; but, again, we must consider how many unknown points will spring up in cases of this kind.

Is gonorrhea in women always a fact—that is to say, a bacteriological fact? Is there any certainty that a fresh infection of the woman comes from the husband? May not the present exacerbation of gonorrhea in the wife antedate to an earlier period, and, in short, is nothing but the old infection brought into activity again?

But especially is it often the case that a woman, two or more years after her married life, will consult a specialist in women's diseases, presenting certain phases of chronic gonorrhea, all symptoms of the disease having long since ceased. The husband, at the same time, consults the andrologist. Is it to be wondered at that there are no gonococci to be found in the husband when they have long since disappeared in the wife? With such uncertain presentations of a case, it is almost impossible to arrive at a satifactory conclusion, and we should not select such cases as examples upon which to base a final judgment.

Generally speaking, then, in cases of gonorrhea in women, how long should one search for the gonococcus in order not to overlook it? That this will happen in many cases, I must not dispute. We cannot question the safe clinical etiological basis created by Wertheim for the diagnosis of gonorrhea, which was accepted by gynecologists long ago. In many cases, especially those presenting a difficult chronic character, it should be proved whether the infection of the urethra, the uterus, etc., originated from the gonorrhea, and, above all, to discover whether the irritating causes of the disease are still present; also, whether the therapy should be regarded as first in order and perhaps alone, or whether we have to deal with the remains of certain disease products, the results of an earlier gonorrheal infection, and, now, whether or not there still remains any virus. In certain cases, these latter and the methods of treatment go hand in hand; but very often it will be necessary to decide what the ultimate outcome of the disease of the urethra, uterus, etc., will amount to, whether any gonococci are still present, or whether there are any cicatricial adhesions, infiltrations and the like. These same conditions are also found in the male, with the exception that the remaining structural changes consequent to a previously existing gonorrhea, long since cured, are more easily kept in abeyance than in case of the woman.

No one will attempt to cure an organic stricture, a cartilaginous epididymis, or perhaps post-gonorrheal nervous affections excited by a diseased urethral mucous membrane, by means of anti-gonorrhoic treatment, if he has not direct proof that the gonococci are still present. It is unfortunate that many gynecologists are loth to accept these points. In the first place, they mistake the earlier manifestations of the disease, with its more or less rightly supposed gonorrheal cause, for later ones, and as a result the woman's suffering is more profound than that of the man; and, secondly, they disregard all those consecutive results whose etiology and course is undoubtedly due to some other affection of the adnexa.

Both of these teachings are unmistakably wrong, and I rejoice at the extraordinary and effective support given these principles, which I have always advocated and upheld, by Dr. Schauta at the gynecologists' congress at Breslau. His arguments were based on sufficient data; he claimed that in inflammatory suppurative diseases of the female adnexa, the gonococcus together with the streptococcus and staphylococcus, conjointly, were, in most instances, the cause of the diseased process; that, even with women who have unquestionably been infected with gonorrhea with subsequent suppurative changes, the latter might have been caused by streptococci; that the clinical progress and the symptoms are not characteristic enough to warrant a differential diagnosis. These opinions of his as regards the diseases of the adnexa led me to the following conclusions, as regards the inflammatory purulent processes of the external genitalia: Without a microscopic examination, a correct diagnosis is impossible, and again, without a correct diagnosis, that is, an etiological diagnosis, there can be no rational therapeutics, because the latter is dependent upon the former, hence very variable.

Dr. Menge, after his investigations at Zweifel's clinic, was also led to the same final conclusions ; that the microscopic proofs of any case showing the undeniable etiology are unconditionally essential as the basis of further therapy.

My honored friend, Sänger, my bitterest opponent in this whole question, nodded contentedly at Schauta's remarks, as if approving a mixed infection. Schauta, if I understand correctly, has not spoken so much about mixed infection—much more of the non-gonorrheic suppurations produced by the streptococci. Mixed infection of gonococci and some of the other pus-producing cocci are not acknowledged by either him or Wertheim, and the suppurative process by mixed infection requires no explanation any more than, as Wertheim has shown, that the gonococci alone may produce suppuration of connective tissue bands, peritonitis, etc. After all, the questions just mentioned, as regards the suppuration in the adnexa, is identical with the question as to the causes of abscesses of Bartholini's glandulæ. These are, indeed, often of gonorrheal origin, but not always (Ulcus molle-infection), and it requires an examination of the pus in order to make an etiologic, differential diagnosis.

I do not deny, however, that by anamnesis, by a careful investigation for gonorrhea in the male, by the appearance of opthalmoblennorrheica in one or more children, and finally, by certain anatomic changes (pointed chondylomata, catarrh of the glands of Bartholini, the macula gonorrheica of the excretory ducts of the same, etc., etc.), one is encouraged to look at once to the diagnosis gonorrhea. I am willing to admit that, in many cases, this supposition is correct, but I deny that with all this status upon which the physician makes his prognosis and therapy, they should not be relied upon ; it should be a settled fact whether there still exists any gonorrhea and are gonococci present, or have we to do with an exterminated gonorrhea, or only with the remnants or consecutive results ? The necessity of this is so much the greater when we find that, with women, all gonorrheal processes, like urethritis, may become chronic.

A physician who is successful in finding the gonococci is in possession of an important positive factor for the future medical progress of the case ; and, naturally, repeated negative results, following microscopic examinations, will, undoubtedly, enable one to handle the case more intelligently as to symptomatic treatment.

I must add a few more words about the significance of the pointed condylomata. I most decidedly oppose the view that there exists any direct relation between these exuberant growths and the gonococci. There might exist one possible relation between the two; that is, in the process of suppuration, the fluor causes maceration and irritation to the mucous membrane. However, many other irritations may produce the same result. The pointed condylomata are not proof positive of the presence of gonorrhea. Where you find pointed condylomata, it is always well to suspect gonorrhea, but to risk any diagnosis on its supposed existence is entirely untrustworthy.

Is there anyone who still denies the importance of searching for the gonococci to support the prophylaxis and therapy? I think none. Therefore, (1) microscopic examinations of the secretions for the gonococci enables us to show, in a great many cases, where macroscopic and clinical means are inefficient, the existence of a still lingering contagion. Through this means we may possibly prevent further spread of the contagion, by constraining prostitutes and others, partly by instructions and partly by force. (2) The therapeutics should not be considered thorough when only certain symptoms of gonorrhea have been removed, *e. g.*, pains, fluor, hemorrhages, etc. The patient should be wholly cured of his gonorrhea, and, therefore, it is advantageous to know whether the therapeutics perfected a complete cure, and whether every trace of the gonococcus has been removed, and this can only be done by a microscopic investigation.

If Broese would only resolve to base his results upon a microscopic research, he would ofttimes observe that there are still gonococci present in the secretions in which he assumed to have destroyed all contagious elements; then, too, he would be convinced that the apparent, or simple, forms of gonorrhea should either be treated lightly or left alone, he probably using, for the destruction of the virus, the most heroic means.

I am convinced that Broese, although attaching little importance to this procedure for the assistance it affords, and the security and certainty it gives to the therapeutics, would not like to see this blessed progress interfered with, the advantages and conveniences of which I and so many colleagues have endeavored to support and advance for years.

### CONCLUSIONS.

1. It cannot be doubted that the gonococcus is the cause of gonorrhea.

2. The diagnosis of gonorrhea in man or woman can, in many cases, be made by observing only the clinical manifestations, without searching for the gonococcus.

3. In many cases, especially those inclined to a chronic course, with few subjective and objective manifestations, the diagnosis is dependent upon a demonstration of the presence of the gonococcus.

4. For this very reason, in all cases of "cures," the investigation for the gonococcus is indispensable to solve the question, whether the discharge is still contagious, or whether we have to deal with only a morbid process, the remains of a former contagion.

5. At all events, the therapy should be based upon the existence or non-existence of the gonococci, and a search for the same should be made not only before, but also during, the whole progress of the treatment.

6. In most cases, microscopic investigation for the gonococci should be sufficient, and, on account of the difficulties which sometimes arise in cultural methods, the progressive treatment is assisted by this means only in particular cases.

7. In every instance where positive results (gonococci) are found, there can be no doubt as to the usefulness and necessity of this means of diagnosis.

Negative results should not be conclusive, because we know of the possibility of gonococci being deeply concealed in the tissue, or in the lamina or folds (invaginations), while at the same time the superficial secretions of the mucous membrane, which we examine, may contain gonococci so few in number as to escape detection. The certainty of their existence, however, will be proven by more frequent examinations for them, and by artificial cultural observations.

The clinical manifestations should always be studied and their relationship strengthened by the use of the microscope.

8. If we have to do with gonorrhea in married people, then both husband and wife should be examined, and, if necessary, both should undergo a course of treatment.

137 West Tupper Street.

